# Generative Adversarial Networks in Brain Imaging: A Narrative Review

**DOI:** 10.3390/jimaging8040083

**Published:** 2022-03-23

**Authors:** Maria Elena Laino, Pierandrea Cancian, Letterio Salvatore Politi, Matteo Giovanni Della Porta, Luca Saba, Victor Savevski

**Affiliations:** 1 Artificial Intelligence Center, Humanitas Clinical and Research Center—IRCCS, Via Manzoni 56, 20089 Rozzano, Italy; pierandrea.cancian@gmail.com (P.C.); victor.savevski@humanitas.it (V.S.); 2 Department of Radiology, Humanitas Clinical and Research Center—IRCCS, Via Manzoni 56, 20089 Rozzano, Italy; letterio.politi@hunimed.eu; 3 Department of Hematology, Humanitas Clinical and Research Center—IRCCS, Via Manzoni 56, 20089 Rozzano, Italy; matteo.della_porta@hunimed.eu; 4 Department of Radiology, University of Cagliari, 09124 Cagliari, Italy; lucasaba@unica.it

**Keywords:** generative adversarial networks, brain imaging, MRI, CT, PET, fMRI

## Abstract

Artificial intelligence (AI) is expected to have a major effect on radiology as it demonstrated remarkable progress in many clinical tasks, mostly regarding the detection, segmentation, classification, monitoring, and prediction of diseases. Generative Adversarial Networks have been proposed as one of the most exciting applications of deep learning in radiology. GANs are a new approach to deep learning that leverages adversarial learning to tackle a wide array of computer vision challenges. Brain radiology was one of the first fields where GANs found their application. In neuroradiology, indeed, GANs open unexplored scenarios, allowing new processes such as image-to-image and cross-modality synthesis, image reconstruction, image segmentation, image synthesis, data augmentation, disease progression models, and brain decoding. In this narrative review, we will provide an introduction to GANs in brain imaging, discussing the clinical potential of GANs, future clinical applications, as well as pitfalls that radiologists should be aware of.

## 1. Introduction

Artificial intelligence (AI) holds significant promise for radiology as it can help in simplifying lesion detection, classification, and segmentation, thereby improving the diagnostic process [1,2,3]. In recent years, Deep Learning (DL) has been proposed and studied in the field of radiology for classification, risk assessment, segmentation tasks, diagnosis, prognosis, and prediction of response to treatment [4].

In 2014, Ian Goodfellow and colleagues developed an innovative Neural Network architecture called Generative Adversarial Networks (GANs). In Goodfellow’s words, “the generative model can be thought of as analogous to a team of counterfeiters, trying to produce fake currency and use it without detection, while the discriminative model is analogous to the police, trying to detect the counterfeit currency. Competition in this game drives both teams to improve their methods until the counterfeits are indistinguishable from the genuine articles” [5].

GANs will be expected to be used in future clinical AI applications since this technology holds a lot of promise for a range of radiological demands [1,6]. Among the many uses of GANs, image-to-image translation and cross-modality synthesis, image reconstruction, image segmentation, image synthesis, data augmentation, disease progression models, and brain decoding are open research fields in radiology. Some of these techniques have already been found useful for different imaging modalities and diseases. Most of the studies have focused on brain disorders, mainly due to the brain’s relatively static and invariable anatomy [7].

In their systematic review, Sorin et al. [1] described the state of the art of GANs’ applications in radiology, analyzing papers published up to September 2019.

Since it represents a very hot topic and research is evolving considerably, in our literature review, we aimed at giving an updated picture of the role that GANs play in the field, with a particular focus on neuroradiology.

## 2. Search Strategy

To perform this narrative review, we selected articles based on our subjective assessment of their relevance to the topic and novelty.

Specifically, we considered as eligible for being included in our review, all the articles that exhaustively answered the following questions—that we considered as driving points to deepen the various sections of our article—:

1. What is a GAN?
2. What are the principal applications of GANs in medical imaging?
3. How are GANs employed in brain imaging?
4. Are there any limitations for GAN in this field?

Secondly, we searched on PubMed the following keywords: “GAN,” “Generative adversarial network”, “brain imaging”, “brain radiology”, “neuroradiology” to retrieve all the papers of our interest. A total of 146 articles were retrieved from this first literature search.

We excluded from our search animals or phantom studies or papers not written in the English language. Furthermore, we decided to exclude all the articles that did not treat any of the topics shown below:

- technical explanation of GANs’ structure;
- focus on at least one application of GANs in brain imaging;
- focus on at least one limitation of GANs in medical imaging.

**Figure 1:**
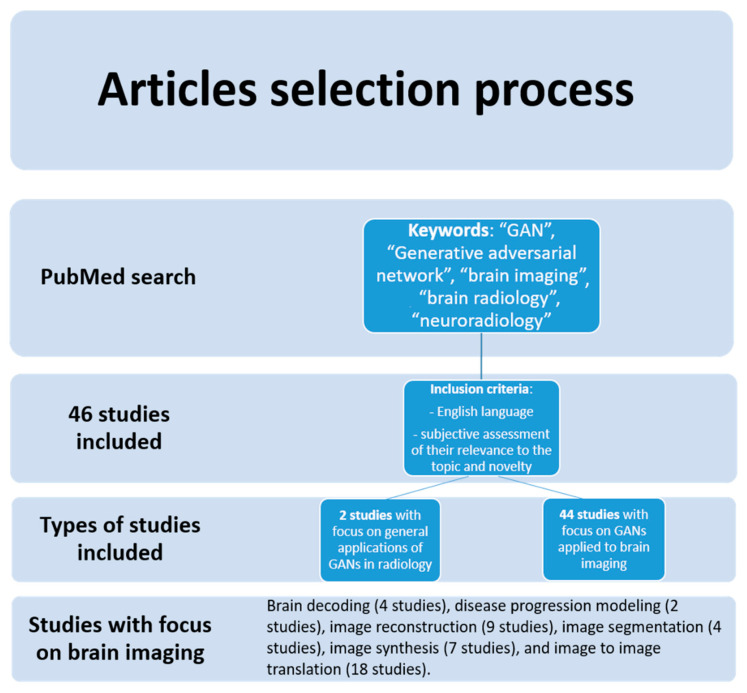
Flow diagram of the study search and inclusion process.

After the subjective screening of all the articles of interest for the aim of our review, two authors who worked independently included and collected data from a total of forty-six studies published between 2018 and 2021. No automation tools were used in this phase. Most of the studies included had a specific focus on a particular application of GANs in brain imaging, such as brain decoding (4 studies), disease progression modeling (2 studies), image reconstruction (9 studies), image segmentation (4 studies), image synthesis (7 studies), and image to image translation (18 studies) (Figure 1).

The main characteristics of the studies included in this review are described in Table 1, Table 2, Table 3, Table 4, Table 5 and Table 6.

## 3. GANs in Brain Imaging

### 3.1. Generative Adversarial Network: A Brief Overview

GANs have been described as an elegant DL approach to generate artificial data that are theoretically indistinguishable from real data. They consist of two networks that work in parallel: a generator G and a discriminator D, which are trained by adversarial learning.

**Figure 2:**
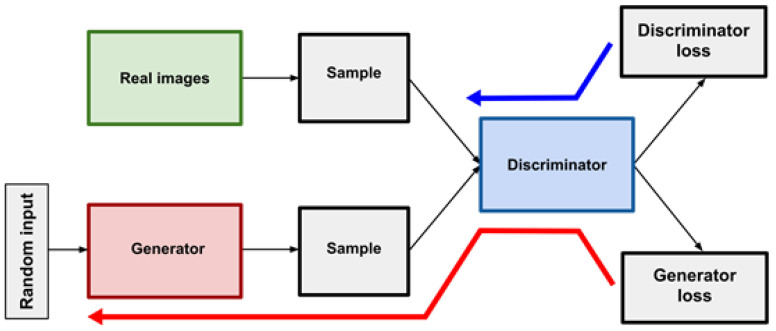
Example of the functioning of a GAN. The generator creates synthetic images from random noise while the discriminator has to differentiate between real and synthetic images. The blue arrow shows the discriminator’s loss back-propagation, the red arrow shows the generator’s loss back-propagation.

As proposed by Goodfellow, a GAN consists of two antagonistic generative neural networks—the generator (G) and the discriminator (D)—pitted against each other with a min-max game mechanism (Figure 2). G creates new data that is then analyzed by D, which has to determine whether the data received was synthetic—generated by G—or real [5].

When building a GAN, D is trained with a dataset of real images, while G—which never saw the training dataset—has to produce images that are very similar to the real ones through the feedback that it receives from D, who “knows” how real images look like.

At the same time, while G creates images, D continuously learns to differentiate between real and generated (fake) images [3]. This process is repeated over and over, so both networks continuously improve their performances, with the ultimate goal of generating images indistinguishable from the real ones.

In this way, the generator will learn to create progressively more realistic images.

In medical imaging, GANs are commonly employed by exploiting either their generative or discriminative nature. Thanks to the former, a GAN learns how to discover the essential structure of training datasets and learns to produce new information, proving to be an essential tool to deal with data scarcity and patient privacy, as they could generate synthetic material to enrich existing datasets or create new anonymous data ex novo.

With regards to their discriminative nature, a GAN can be employed as a “regularizer” or “detector” of aberrant images since the discriminator D learned in the training phase how a normal image is made, and it can recognize all the images that significantly differ from the ones it already “saw”.

Since 2014, GANs have evolved in their architectural design, leading to the creation of new models such as Conditional Generative Adversarial Networks (CGAN), Deep Convolutional Generative Adversarial Networks (DCGAN), Wasserstein Generative Adversarial Networks (WGAN), and many more.

Evaluation of the performance of GANs is still a hot topic, and an optimal numerical metric to assess the quality of the synthetic images produced by the generator hasn’t been proposed yet. This is strongly related to the absence of an objective loss during the generator training.

The evaluation can be done through qualitative or quantitative metrics. It is important to keep in mind that there is no consensus as to which measure best captures the strengths and limitations of models and should be used for fair model comparison [8]. The Inception Score (IS), one of the most widespread metrics, is based on the use of a pretrained InceptionNet (hence the name) to classify the generated images into different classes. The score rises if the images are highly classifiable and diverse. The Fretchet Inception Distance (FID) was introduced by [9] and worked by computing the distance between real and generated images. The data is embedded into a feature space through a CNN (often an InceptionNet), and a multivariate Gaussian is used to approximate the embeddings; the distance is calculated on the mean and covariance. Other metrics are based on the quality of the image itself: the Structure Similarity Index Measure (SSIM) the Peak Signal-to-Noise Ratio (PSNR) are common examples. The SSIM and its multi-scale variant (MS-SSIM) describe the similarity of paired images at the structure level, operating on corresponding pixels and their neighbors. The PSNR is used to compute the amount of noise between paired images.

### 3.2. Applications of GAN in Brain Imaging

The most important brain imaging techniques include computed tomography (CT), magnetic resonance imaging (MRI), functional magnetic resonance imaging (fMRI), and positron emission tomography (PET) [6]. Each of them provides important reference information regarding early diagnosis, identification, treatment, and follow-up of the disease.

Radiologists have been applying AI to a wide range of tasks, and, specifically for brain imaging, DL has mainly been used for the detection, classification, segmentation of anatomical structures and lesions [10,11]. With the advent of GANs, other scenarios are opening in this field, resulting in a direct positive impact in patients’ care by, among others, lowering radiation and contrast dose, shortening examinations’ time, and reducing costs [12].

In particular, the main applications of GANs in brain imaging are:

- Image-to-image translation and cross-modality synthesis;
- Image reconstruction: Super-resolution and artifact removal;
- Image Segmentation;
- Image Synthesis and data augmentation;
- Disease progression modeling;
- Brain decoding.

#### 3.2.1. Image-To-Image Translation and Cross-Modality Synthesis

One of the most interesting applications of GANs is their ability to translate data between different techniques, as they can “translate” images across different modalities—for example, transforming CT to MR or PET images and vice versa (cross-modality synthesis) [13]—or generate new images in the setting of the same modality—for example transforming images across different MRI sequences from T1-weighted to T2-weighted—image-to-image translation) [13]. This could bring a significant reduction of acquisition times or radiation exposure and prevent patients from undergoing multiple examinations. Furthermore, in this setting, GANs could be particularly useful in cases where there is limited access to different imaging devices when the patients need to lie still for a long time and for reducing costs [1].

Moreover, the possibility of having access to multi-modality information could help doctors make a more accurate diagnosis [14] and ease their work, as collecting data from the same patient using different imaging techniques is often impractical [15].

Table 1 provides a summary of the papers included in the review, focused on GANs applications in image-to-image translation and cross-modality synthesis.

Jin et al. [16] used a CycleGAN-like approach, called MR-GAN, to convert CT into MR images for radiotherapy planning. The authors focused mainly on patients for which MR was not recommended due to claustrophobia or the presence of cardiac pacemakers, as well as other scenarios in which only CT scans were available. The authors advocate for the use of both paired and unpaired data, reaching better results in terms of MAE, SSIM, and PSNR than when using only one kind. Leveraging unpaired data is crucial in medical image translation, as acquiring both CT and MR images of a single patient in a short time span is often infeasible and puts a hard limit on the available data. 

Kazemifar et al. [17] used GAN to generate brain CT images from MR images of patients affected by brain tumors for treatment planning (intensity-modulated proton therapy), obtaining excellent dosimetric accuracy. Similarly, Maspero et al. [18] included 60 pediatric patients undergoing brain radiotherapy. The authors trained three 2D CGANs to generate synthetic CT (sCT) from T1-weighted MRI. They were able to obtain accurate MR-based dose calculations. Liu et al. [19] and Yang et al. [20] used GAN to synthesize CT images from T1-weighted MRI showing promising results for dosimetric accuracy.

Lan, Toga, and Sepehrband [21] propose a new GAN pipeline that is designed and optimized for the application of multimodal 3D neuroimaging synthesis. They first expanded the original conditional GAN architecture to 3D space by using 3D (transposed) convolutions and introduced an array of stabilization techniques to ensure the training procedure remained smooth. The performance of the network was evaluated on the data set from ADNI-3, in which the proposed network was used to predict PET images, fractional anisotropy, and mean diffusivity maps from multimodal MRI. The prediction error of the SC-GAN was 18%, 24%, and 29% lower compared to 2D conditional GAN for fractional anisotropy, PET, and mean diffusivity tasks, respectively.

Recently, since a new class of specialized stand-alone PET scanners has been introduced, there is a need for accurate methods to perform attenuation correction. These new PET scanners are low-cost, specialized in brain imaging, and have a high spatial resolution [13]. Attenuation correction (AC) is necessary to estimate radiotracer distribution in PET [22].

Armanious et al. [13] estimated attenuation maps from non-attenuation corrected data by using GANs. During their experiment, the generator received a non-attenuation corrected PET image and synthesized a pseudo-CT image resulting in independently attenuation-corrected PET data. They showed no regional bias and only minimal average errors, around ±0% in the attenuation maps, and no differences in image-based diagnoses in 20 patients with neurological disorders.

Similarly, Gong et al. [23] showed that Cycle-GAN could generate AC comparable performance to standard CNN-based pipelines in their series—32 patients (14 males, 18 females) who underwent 18F-FDG PET/CT and PET/MRI without pathology. The proposed methods created synthetic CTs for AC computation. Its performance in terms of the Dice score of the bone region outclassed the ones based on the more conventional Atlas or Segmentation method.

#### 3.2.2. Image Reconstruction: Super Resolution and Artifact Removal

GANs find another interesting application in the modification of acquired images by improving both the definition and the quality and reducing artifacts.

With SR techniques, GANs generate high-resolution (HR) from low-resolution (LR) images. Furthermore, GANs can also remove artifacts in the area under investigation, thus avoiding an important reduction in image quality, which negatively influences radiological interpretation.

Super-resolution techniques, often known as simply super-resolution, allow to increase the resolution of an image and thus its quality. Traditionally, SR was done through standard image processing techniques, but recently GANs have shown that they can be a powerful tool in the task of SR. SR is especially interesting in the medical field as the low resolution can be an intrinsic characteristic of the imaging modality, such as in the case of PET, or can be related to the acquisition process, such as in the case of low field MR scanners.

By improving the quality of images, GANs can be useful in the post-processing of examinations like low dose scans or images containing artifacts—thus preventing patients from undergoing additional or repeated examinations—and in reducing the dose of radiation administered.

Artifacts represent one of the main limitations in imaging. They are usually caused by motion, or the presence of metallic materials in the region examined. Concerning MRI, motion can be classified into two categories [24]:

- Rigid motion, which is caused by the movement of a solid part of the body, in which deformation is zero or so small it can be neglected, such as arm, knee, and head motion; and
- Non-rigid motion, which arises from those parts of the body that do not retain any consistent shape, like cardiac motion.

Regarding intra-brain scans, the contribution of rigid motion is more significant in contrast to non-rigid motion. For this reason, one of the challenges of brain imaging is that it depends on the patients’ ability to remain still. GANs can improve the quality of images by removing artifacts, and this could be especially useful for patients who cannot lie still for long periods.

Table 2 provides a summary of the papers included in the review, focused on GANs applications in image reconstruction.

Ouyang et al. [25] raised the issue that obtaining HR PET images usually implies exposing the patient to a higher radiation dose. For this reason, they used a GAN to synthesize standard-dose amyloid PET images from ultra-low-dose images. They used 40 PET datasets from 39 participants with a simultaneous PET/MRI scanner after an injection of 330 ± 30 MBq of the amyloid radiotracer, 18F-florbetaben. They were able to synthesize standard-dose amyloid PET images from ultra-low-dose images with an image quality score of 4.27 ± 0.56. Their GAN [25] outperformed a previously developed CNN [26] that required additional MRIs.

Zhao et al. [27] used a supervised deep learning approach to create a GAN, named S-CycleGAN, obtained by a fusion of the cycle-consistency loss, Wasserstein distance loss, and an additional supervised learning loss. Their aim was to establish a non-linear end-to-end mapping model to recover low-dose PET (LDPET) brain images. For this purpose, they selected 109 patient PET/CT scans acquired with the injection of 370.81 ± 64.38 MBq of 18F-fluorodeoxyglucose (FDG). Their model was able to suppress image noise and preserve structure details in a supervised learning fashion with an SSIM of 0.981 ± 0.00803 and 0.994 ± 0.00262 for 10% and 30% doses, respectively.

Song et al. [22] focused on resolution enhancement using an unsupervised SR method based on dual GAN to generate SR brain PET images. The network was trained using unpaired clinical LR and HR PET images, the latter acquired with an HR dedicated brain PET scanner. The LRPET images corresponded to an older scanner model. They obtained an SSIM of 0.926 and demonstrated promising results in terms of image quality.

Furthermore, CGAN has aided motion correction of involuntary subject motion during dynamic 18F-FDG brain studies. Sundar et al. [28] developed a fully automated motion compensation approach that enabled the translation of non-invasive clinical absolute quantification from PET/MR to PET/CT [28] using 20 brain images taken from 10 healthy volunteers who underwent a test-retest 18F-FDG PET/MRI examination.

Zhou et al. [12] trained a GAN model to learn from 1.5-T and 3-T scans obtained from the same subjects at the same time to generate 3T* synthetic images, jointly training a Fully Convolutional NN (FCN) to detect Alzheimer’s Disease in the generated 3T* images. The FCN classification loss propagated back to implicitly convey the disease-related information to the generator, which then created 3T* images where the Alzheimer’s Disease was more evident.

Delannoy et al. [29] discussed a GAN-based framework (SegSRGAN) that performed SR as well as cortical segmentation of neonatal brain MR. They used two datasets for a total of 1540 LR images. Their model obtained a Dice Score of 0.845 ± 0.015 regarding the cortical segmentation, while the SR scored the following: NCC 3.87 ± 2.47, PSNR 26.84 ± 0.62, and SSIM 0.721 ± 0.012.

Shaul et al. [30] proposed a solution to reduce the acquisition time of MRI examinations, making a point that reducing the number of samples in the k-space was a very effective way to speed up the acquisition time. They employed a two-stage GAN to estimate missing k-space samples and reduce aliasing artifacts, obtaining an SSMI of 0.98 ± 0.01.

More recently, Zhang, Shinomiya, and Yoshida [31] successfully combined two DCGAN to perform 3D reconstruction of MRI images in a 2D field, solving artifacts and color changes caused by the checkerboard effect. The results show their model had the best performance for all the planes, with an SSIM of 0.9201 ± 0.0041, 0.9641 ± 0.0032, and 0.9600 ± 0.003 for the sagittal, coronal, and axial planes, respectively.

#### 3.2.3. Image Segmentation

In brain imaging, image segmentation finds its main applications in measuring and visualizing anatomical structures, analyzing brain changes, delineating pathological regions, and for surgical planning and image-guided interventions [10] or for the training of other DL algorithms, saving time and resources of radiology professionals and optimizing costs.

However, GANs have not been widely used for segmenting images on their own, but they can help obtain a better segmentation quality if used along with other DL algorithms that can perform supervised, semi-supervised or automatic segmentation [10].

For example, a CNN—which represents a standard network to perform segmentation—can be used as the generator and be trained with adversarial learning. At that point, the discriminator takes as input the segmentation mask produced by the CNN and the ground truth image and has to discriminate between the two. This process, repeated over and over, will improve the performances of the CNN—used as a generator—in its segmentation task.

Furthermore, a GAN can augment the initial dataset of ground truth images [10] used to train a CNN, enriching the data from which the CNN learns and thus helping it to reach better performances.

Moreover, a GAN could be useful for “domain adaptation” [10] also in this case.

For example, if trained with segmented T1w MRI images, the GAN is able to generate the corresponding T2w image, which contains the segmentation mask in the same location as the ground truth images.

Table 3 provides a summary of the papers included in the review, focused on GANs applications in image segmentation.

Oh et al. [11] used a GAN to segment white matter from brain PET images to improve the diagnosis of neurodegenerative disorders. To train their model, based on the pix2pix architecture with a ResNet backbone, the authors used 192 18F-FDG PET/CT and MRI images collected from the Alzheimer’s disease neuroimaging initiative (ADNI) database, obtaining an AUC mean value of 0.869 ± 0.021.

Yuan et al. [10] proposed a 3D unified GAN, which unifies the any-to-any modality translation and multimodal segmentation in a single network brain tumor segmentation obtaining a dice of 0.5089 and sensitivity of 0.5290 and specificity of 0.9987.

Delannoy et al. [29] proposed a GAN-based framework (SegSRGAN) that jointly performed SR and segmentation, as opposed to the more common sequential route. They argued that performing these tasks jointly improved the performance of both the segmentation and the reconstruction process, reporting a dice score of 0.855 ± 0.014.

#### 3.2.4. Image Synthesis and Data Augmentation

Artificial intelligence models have shown great promise in assisting clinicians by automating the diagnostic process; however, to achieve the appropriate performance, the models must be trained with a large amount of data. Unfortunately, this represents a challenge in the medical field, mostly due to the high standards of privacy that hinder the possibility of creating large open datasets to share with the scientific community.

Indeed, the scarcity of data represents one of the main limitations to the application of DL in medicine (research) [32,33]. Moreover, the respect for anonymity and privacy can limit the use of patients’ data for research purposes when informed consent is not obtained [34].

GANs have the ability to create new data not belonging to any real person, bypassing all the privacy and anonymity concerns that can be used in various clinical applications [35]. Usually, translation, rotation, scaling, and flipping of available samples to generate “new” samples are common strategies for data augmentation. These kinds of deterministic data augmentation techniques, while effective, are under tighter constraints in the medical field: because medical images cannot be distorted in shape and color, their applicability is limited. GANs allow the synthesis of completely new scans to enlarge datasets by generating images that have all the characteristics of the real images from real patients [3]. Table 4 provides a summary of the papers included in the review, focused on GANs applications in image synthesis and data augmentation.

In 2018, Kazuhiro et al. [7] used a GAN network (DCGAN) to produce artificial T1-weighted MR brain images from healthy individuals and patients with cerebrovascular accidents. Their performance was rated by two neuroradiologists who tried to discern the synthetic images from the real ones: 45% and 71% of the synthetic images were rated as real MRIs by each radiologist, respectively, while 44% and 70% of the real images were rated as synthetic images.

Likewise, Li et al. [36] applied GANs to augment brain data sets by generating paired data which were used to train and test different DL-based segmentation techniques.

Kim et al. [37] used a model called Boundary Equilibrium Generative Adversarial Network (BEGAN) to extract features of Alzheimer’s disease and normal cognitive brain 18F FDG PET/CT. They then used these features to train a support vector machine (SVM) classifier to distinguish brains affected by Alzheimer’s disease from the normal cognitive brain.

Islam and Zhang [32] used DCGAN to produce synthetic PET images of three different stages of Alzheimer’s disease, showing that synthesized brain images share nearly identical data and sharpness as the original real dataset.

#### 3.2.5. Brain Decoding

Reading minds has been part of works of fiction.

However, in recent years, breakthroughs in neuroscience and AI have brought to reality the possibility of visual image reconstruction decoding, providing intuitive and vivid pictures regarding the objects a person is viewing [38,39]. GAN networks have been used to achieve the accurate reconstruction of natural images from brain activity by measuring fMRI brain activation patterns for decoding the identity of binary contrast patterns, handwritten characters, human facial images, and even dreams.

GANs may analyze fMRI-derived brain activity in two steps. A visual stimulus is first processed via neural networks to form a latent representation (code) of itself. This is then received by a decoder sub-network, which seeks to reconstruct and reproduce the visual input from the data in the code [4,40]. Table 5 provides a summary of the papers included in the review, focused on GANs applications in brain decoding.

Ren et al. [38] reconstructed geometric shapes, alphabet letters, and natural color photos from brain activity by visually-guided cognitive representation and adversarial learning, obtaining 89% identification accuracy. Huang et al. [39] proposed a DL-based framework that included a latent feature extractor, a latent feature decoder, and a natural image generator to achieve the accurate reconstruction of natural images from brain activity. Their results showed that the reconstructed image achieved comparable, accurate reproduction of the presented image in both high-level semantic category information (which refers to the image context as perceived by humans) and low-level pixel information (related to lower pixel dimensions and thus better image quality).

Al-Tahan and Mohsenzadeh [40] realized a GAN made up of two sub-networks (encoder-decoder) that mimicked the human brain temporal (obtained by magnetoencephalography—MEG) and spatial (obtained by fMRI) response patterns in biological vision.

Qiao et al. [4] proposed a new GAN-based Bayesian visual reconstruction model (GAN-BVRM) composed of (1) a classifier to decode the categories from fMRI data, (2) a pre-trained conditional generator of the distinguished BigGAN to generate natural images for the categories, and (3) a set of encoding models and an evaluator to evaluate the generated images. Their model was able to directly generate the reconstructed images by iteratively updating the noise input vector through back-propagation to fit the fMRI voxels and improved the fidelity and naturalness.

#### 3.2.6. Disease Progression Modelling

Thanks to technological developments, much progress has been made in terms of outcome prediction through the generation of several machine learning-based prognostic models. Historically most of the models to estimate the progression of disease were based on systems of a partial differential equation that require careful tuning and cannot learn from the available data.

In this setting, GANs have been successfully employed in brain tumor growth prediction and anomaly detection. Indeed, some authors succeeded in the generation of the possible images of future MRI scans of a patient by training the GAN with the current MRI examinations.

Table 6 provides a summary of the papers included in the review, focused on GANs applications in Disease Progression Modelling.

Elazab et al. [6] developed a 3D U-Net architecture, named GP-GAN, that showed outperforming results for growth prediction of glioma compared to state-of-the-art methods, reaching a sensitivity of 92.06 ± 1.46 and a specificity of 91.27 ± 2.12. Han et al. [41] developed a two-step unsupervised medical anomaly detection generative adversarial network (MADGAN) to discriminate subtle lesions on brain MRI in patients with early-stage Alzheimer’s disease and brain metastasis, which helped in establishing a disease progression model. They were able to detect Alzheimer’s disease on T1 scans at a very early stage, with AUC 0.727, and at a late stage with AUC 0.894, while detecting brain metastases on T1c scans with AUC 0.921.

## 4. Discussion

GANs represent very promising tools that could help doctors and health professionals in their clinical and research routine from many points of view.

One of the major benefits of GANs is related to their content generation ability, which could help in coping with the lack of data, anonymity, and quality of data [6]. Furthermore, the possibility for image-to-image translation and cross-modality synthesis can be of benefit for both patients and radiologists as it may reduce radiation exposure and improve image interpretation [34]. With regards to image reconstruction, studies have shown how GANs can reduce CT radiation exposure and MRI acquisition time, which may influence screening and follow-up protocols and the radiologist workload. Moreover, GANs allow improving data availability and quality at a low cost, which could be of great help for underdeveloped and developing countries that usually do not have high-quality scanners or where patients do not have access to some type of examinations. Indeed, as explained above, by using a GAN, a low-resolution CT scan can be transformed into a high-resolution one or even in an MR examination. As it is well known, DL algorithms require large-scale data sets, which can sometimes be difficult to obtain, especially for rare diseases limiting the development and implementation of DL in radiology. Current advances in this aspect show promising results as GANs can help overcome this obstacle. Indeed, several studies have successfully trained deep learning algorithms using synthetic data generated by GANs [1,2].

GANs have evolved considerably since their introduction, but even if there is hype in the research field, they are still not applied in clinical practice. One reason that GANs remain in a proof-of-concept stage is that their successful development and training can be difficult.

The correct functioning of a GAN implies the harmonic combination between the generator and the discriminator. An interruption of their balance can make the training process fail. Another problem commonly presented is the complete or partial mode collapse. In this case, the generator will synthesize a limited diversity of images or even the same image, regardless of its input. In radiology, this can cause the generation of wrong artificial features. The networks can also be biased when there is under or over-representation of certain findings. This could lead to misdiagnosis of medical conditions as the composition of the source and target domains can bias the image transformation to cause an unwanted feature hallucination [42].

Furthermore, the type and the quality of the new data generated by GANs are strictly dependent on the training dataset [1]. If a GAN is trained to generate brain images, it will earn how to create new scans based on the images “saw” in the training phase. For this reason, if it was trained with a dataset of adults’ brain MRIs, the GAN would not be able to generate MRI images of a pediatric brain. Thus, it is important to determine the purpose of each GAN, design data flow, and train it with large data sets.

Some other risks are involved with the development of GANs. For example, in image reconstruction, details can get lost in translation, while fake inexistent details can suddenly appear [1]. Furthermore, the possibility of hacking of imaging examinations and artificially adding or removing medical conditions from patient scans.

Moreover, hackers could infiltrate the health care system with malicious intent, and the effects of such attacks have already been shown. Mirsky et al. [43] manipulated CT scan images by artificially adding or removing lung cancers on purpose, showing those images to radiologists, who were blinded to the attack. The hack showed a 99.2% success rate for cancer injection and 95.8% for removal. However, even when the radiologists were informed of the attack, the success rates were still high (70% for cancer injection decreased and 90% for cancer removal). The consequences of these types of attacks could be dangerous, and they could be addressed to a specific patient or an institution.

Although their interesting and sophisticated structure, GANs are significantly subjected to validation problems. Indeed, it is difficult to assess whether generated data can be considered as “reliable”, in the sense that they’re sufficiently similar to real ones.

While this could be relatively easy for imaging, where generated data can be validated through visualization, a standardized metric of validation has to be implemented when considering tabular or time-series data. For this reason, some alternative generative deep learning frameworks have been proposed, with the main one being Variational autoencoders (VAEs) [44,45].

Autoencoders are a very popular deep learning framework in which data are “mapped” (projected) to a lower-dimensional latent space by a first neural network, the encoder, and then reconstructed by a second one, the decoder. The two networks are jointly trained by gradient descent to minimize the reconstruction loss between original and reconstructed data. Once the model has been trained, the latent space representation can maintain much of the information carried by the original data and can be used for a series of tasks such as dimensionality reduction or anomaly detection [46].

The problem with this kind of architecture lies in the way the latent space is structured. Indeed, the network has to learn the latent representation of each data point, with no relevant constraint on the representation itself. This can result in high training time and overfitting and doesn’t provide a probabilistic framework suitable for generative tasks.

VAEs have been presented as the solution to these issues. Indeed, in these models, Bayesian variational inference [47] is used to learn the parameters of a latent distribution (usually a Gaussian), and then data points are sampled from the learned distribution and used to generate reconstructed data. More in detail, the encoder is equipped with additional dense layers representing the parameter of the target distribution (mean and standard deviation for a Gaussian), and a reparametrization trick is used to minimize the Evidence Lower Bound (ELBO) between the encoded data and a given prior distribution through back-propagation. This bayesian “comparison” between the encoded data and the prior provides a strong regularization on the latent space, making sure that the model very rarely overfits the data and, by the sampling procedure carried on by the decoder, enabling the generative framework.

Focusing on imaging application of VAEs, it is straightforward to see that the reconstruction loss framework ensures a different generative process: indeed, since the latent distribution is learned in the process, generated data must more strongly rely on training samples and the generative part, the decoder, is constrained to follow that latent distribution when generating new images [45]. In other words, while in GANs, the generator is left free to learn a distribution from random noise to fool the discriminator, in VAEs, the decoder is forced to rely on a learned latent space.

In conclusion, images generated by GANs remain overall more detailed than the ones generated from VAEs, which tend to be “blurry”. Considering this, if GANs can be efficiently validated, they tend to generate better quality images than VAEs.

This review has several limitations. First of all, we designed it as a narrative review to make an overview of what state of the art is on the topic. Thus, the literature search has not been conducted in a systematic fashion.

Secondly, effective assessment and comparison of GANs’ performances were difficult between the considered articles. That is due to the variability of assessment measures between studies. Some use objective numerical metrics of different sorts, but those vary between studies. Examples include mean absolute error (MAE), peak signal-to-noise ratio (PSNR), structural similarity (SSIM), Area Under Curve (AUC), Frechet Inception Distance (FID). This lack of standardization and the use of subjective physicians’ evaluations of image quality prevented us from performing a meta-analysis. Furthermore, the exact number of patient cases was not clear in some of the papers.

Finally, the studies included in this review lack four fundamental criteria for algorithm’s clinical assessment described by Kim et al. [37]: external validation, prospective data collection, data being obtained in a diagnostic cohort study, and from multiple institutions. The reviewed articles were new in each subfield of medical imaging and used small data sets. Furthermore, most of the published studies ultimately assess technical feasibility but not the practical clinical performance of GANs in radiology.

## 5. Conclusions

In conclusion, GANs in brain imaging show great potential, which is reflected in the increasing number of studies. They enable the creation of new data or the imaging modality transfer, which can be useful for several purposes. The achievement of low-dose imaging and shortening acquisition time while maintaining image quality or improving the image quality by removing artifacts certainly are of clinical relevance. Yet, it is still early to determine whether GANs can significantly impact computer-assisted radiology applications. More studies are needed, preferably with larger datasets, in order to determine the feasibility of GANs in the clinical setting.

## Figures and Tables

**Table 1:** Articles included in the review focusing on image-to-image translation and cross-modality synthesis.

Author	Year	Application	Population (No. of Patients)	Imaging Modality	ML Model	Results
Jin	2019	Image-to-Image translation and cross-modality synthesis	202 patients	MRI from CT image	MR-GAN	MAE: 19.36 PSNR: 65.35 SSIM: 0.25
Kazemifar	2019	Image-to-Image translation and cross-modality synthesis	66 patients	CT from MRI	GAN	mean absolute difference below 0.5% (0.3 Gy)
Dai	2020	Image-to-Image translation and cross-modality synthesis	274 subjects (54 patients with low-grade glioma, and 220 patients with high-grade glioma)	MRI	multimodal MR image synthesis method unified generative adversarial network.	NMAEs for the generated T1c, T2, Flair: 0.034 ± 0.005, 0.041 ± 0.006, and 0.041 ± 0.006. PSNRs: 32.353 ± 2.525 dB, 30.016 ± 2.577 dB, and 29.091 ± 2.795 dB. SSIMs: 0.974 ± 0.059, 0.969 ± 0.059, and 0.959 ± 0.05.
Hamghalam	2020	Image-to-Image translation and cross-modality synthesis	Various datasets	MRI-HTC	Cycle-GAN	Dice similarity scores: 0.8%, (whole tumor) 0.6% (tumor core) 0.5% (enhancing tumor).
Maspero	2020	Image-to-Image translation and cross-modality synthesis	60 pediatric patients	SynCT from T1-weighted MRI	cGANs	mean absolute error of 61 ± 14 HU pass-rate of 99.5 ± 0.8% and 99.2 ± 1.1%
Sanders	2020	Image-to-Image translation and cross-modality synthesis	109 brain tumor patients	relative cerebral blood volume (rCBV) maps from computed from DSC MRI, from DCE MRI of brain tumors	cGANs	Pearson correlation analysis showed strong correlation (ρ = 0.87, *p* < 0.05 and ρ = 0.86, *p* < 0.05).
Wang	2020	Image-to-Image translation and cross-modality synthesis	20 patients	MRI-PET	cycleGANs	PSNR > 24.3 SSIM > 0.817 MSE ≤ 0.036.
Lan	2021	Image-to-Image translation and cross-modality synthesis	265 subjects	PET-MRI	3D self- attention conditional GAN (SC- GAN) constructed	NRMSE:0.076 ± 0.017 PSNR: 32.14 ± 1.10 SSIM: 0.962 ± 0.008
Bourbonne	2021	Image-to-Image translation and cross-modality synthesis	184 patients with brain metastases	CT-MRI	2D-GAN(2D U-Net)	mean global gamma analysis passing rate: 99.7%
Cheng	2021	Image-to-Image translation and cross-modality synthesis	17 adults	Two-dimensional fMRI images using two-dimensional EEG images;	BMT-GAN	MSE: 128.6233 PSNR: 27.0376 SSIM: 0.8627 VIF: 0.3575 IFC: 2.4794
La Rosa	2021	Image-to-Image translation and cross-modality synthesis	12 healthy controls and 44 patients diagnosed with Multiple Sclerosis	MRI (MP2RAGE uniform images (UNI) from MPRAGE)	GAN	PSNR: 31.39 ± 0.96 NRMSE: 0.13 ± 0.01 SSIM: 0.98 ± 0.01
Lin	2021	Image-to-Image translation and cross-modality synthesis	AD 362 subjects; 647 images CN 308 subjects; 707 images pMCI 183 subjects; 326 images sMCI 233 subjects; 396 images	MRI-PET	Reversible Generative Adversarial Network (RevGAN)	Synthetic PET: PSNR: 29.42 SSIM: 0.8176 PSNR: 24.97 SSIM: 0.6746
Liu	2021	Image-to-Image translation and cross-modality synthesis	12 brain cancer patients	SynCT images from T1-weighted postgadolinium MR	GAN model with a residual network (ResNet)	Average gamma passing rates at 1%/1 mm and 2%/2 mm were 99.0 ± 1.5% and 99.9 ± 0.2%,
Tang	2021	Image-to-Image translation and cross-modality synthesis	37 brain cancer patients	SynCT from T1-weighted MRI	GAN	Average gamma passing rates at 3%/3 mm and 2%/2 mm criteria were 99.76% and 97.25%
Uzunova	2021	Image-to-Image translation and cross-modality synthesis	Various datasets	MRI (T1/Flair to T2, healthy to pathological)	GAN	T1 → T2 SSIM: 0.911 MAE: 0.017 MSE: 0.003 PSNR: 26.0 Flair → T2 SSIM: 0.905 MAE: 0.021 MSE: 0.004 PSNR: 24.6
Yang	2021	Image-to-Image translation and cross-modality synthesis	9 subjects	Multimodal MRI-CT registration into monomodal sCT-CT registration	CAE-GAN	MAE: 99.32

**Table 2:** Articles included in the review focusing on image reconstruction.

Author	Year	Application	Population (No. of Patients)	Imaging Modality	ML Model	Results
Ouyang	2019	Image reconstruction	39 participants	PET/MRI	GAN	MAE: 8/80
Song	2020	Image reconstruction	30 HRRT scans from the ADNI database. Validation dataset = 12 subjects	low-resolution PET and high-resolution MRI images	Self-supervised SR (SSSR) GAN	Various results
Shaul	2020	Image reconstruction	490 3D brain MRI of a healthy human adult; 64 patients from Longitudinal MS Lesion Segmentation Challenge (T1, T2, PD, and FLAIR); 14 DCE-MRI acquisitions of Stroke and brain tumor	MRI	GAN	PSNR: 40.09 ± 3.24 SSIM: 0.98 ± 0.01 MSE: 0.0021 ± 0.036
Zhao	2020	Image reconstruction	109 patients	PET	S-CycleGAN	Average coincidence: 110 ± 23
Zhang	2021	Image reconstruction	581 healthy adults	MRI	noise-based super-resolution network (nESRGAN)	SSIM: 0.09710 ± 0.0022
Sundar	2021	Image reconstruction	10 healthy adults	PET/MRI	cGAN	AUC: 0.9 ± 0.7%
Zhou	2021	Image reconstruction	151 patients with Alzheimer’s Disease	MRI	GAN	Image quality: 9.6%
Lv	2021	Image reconstruction	17 participants with a brain tumor	MRI	PI-GAN	SSIM: 0.96 ± 0.01 RMSE: 1.54 ± 0.33
Delannoy	2020	Image reconstruction and segmentation	dHCP dataset = 40; Epirmex dataset = 1500	MRI	SegSRGAN	Dice 0.050

**Table 3:** Articles included in the review focusing on image segmentation.

Author	Year	Application	Population (No. of Patients)	Imaging Modality	ML Model	Results
Liu	2020	Image segmentation	14 subjects	MRI	cycle-consistent generative adversarial network (CycleGAN)	Dice 75.5%; ASSD: 1.2
Oh	2020	Image segmentation	192 subjects	18 F-FDG PET/CT and MRI	GAN	AUC-PR: 0.869 ± 0.021
Yuan	2020	Image segmentation	484 brain tumor scans	MRI	GAN	Dice: 42.35%

**Table 4:** Articles included in the review focusing on image synthesis.

Author	Year	Application	Population (No. of Patients)	Imaging Modality	ML Model	Results
Kazuhiro	2018	Image synthesis	30 healthy individuals and 33 patients with cerebrovascular accident	MRI	DCGAN	45% and 71% were identified as real images by neuroradiologists.
Islam	2020	Image synthesis	479 patients	PET	DCGAN	SSIM 77.48
Kim	2020	Image synthesis	139 patients with Alzheimer’s Disease and 347 Normal Cognitive participants.	PET/CT	Boundary Equilibrium Generative Adversarial Network (BEGAN)	Accuracy: 94.82; Sensitivity: 92.11; Specificity: 97.45; AUC:0.98
Qingyun	2020	Image synthesis	226 patients (HGG (high-grade gliomas): 166, LGG (low-grade gliomas): 60) as a training set to train TumorGAN,	MRI (FLAIR, T1, T1CE)	TumorGAN	Dice 0.725
Barile	2021	Image synthesis	29 relapsing-remitting and 19 secondary-progressive MS patients.	MRI	GAN AAE	F1 score 81%
Hirte	2021	Image synthesis	2029 patients normal brain	MRI	GAN	Data similarity 0.0487
Kossen	2021	Image synthesis	121 patients with Cerebrovascular disease	MRA	3 GANs: (1) Deep convolutional GAN, (2) Wasserstein-GAN with gradient penalty (WGAN-GP), and (3) WGAN-GP with spectral normalization (WGAN-GP-SN).	FID 37.01

**Table 5:** Articles included in the review focusing on brain decoding.

Authors	Year	Application	Population (No. of Patients)	Image Modality	ML Model	Results
Qiao	2020	Brain decoding	1750 training sample and 120 testing sample	fMRI	GAN-based Bayesian visual reconstruction model (GAN-BVRM)	PSM: 0381 ± 0.082
Ren	2021	Brain decoding	Various datasets	MRI	Dual-Variational Autoencoder/Generative Adversarial Network (D-Vae/Gan)	Mean identification accuracy: 87%
Huang	2021	Brain decoding	Five volunteers (3 males and 2 females)	fMRI	CAE, LSTM, and conditional progressively growing GAN (C-PG-GAN) deep	Various results for each participant
Al-Tahan	2021	Brain decoding	50 healthy right-handed participants	fMRI	Adversarial Autoencoder (AAE) framework	MAE 0.49 ± 0.024

**Table 6:** Articles included in the review focusing on disease progression modeling.

Author	Year	Application	Population (No. of Patients)	Imaging Modality	ML Model	Results
Elazab	2020	Disease progression modeling	9 subjects	MRI	growth prediction GAN (GP-GAN) GP-GAN	Dice: 88.26 Jaccard Index: 78.99
Han	2021	Disease progression modeling	408 subjects/1133 scans/57,834 slices	MRI	medical anomaly detection generative adversarial network (MADGAN)	Cognitive impairment: AUC: 0.727 AD late stage: AUC 0.894 Brain metastases: AUC 0.921
